# Quo Vadis, Dottore? Religious, Philosophical and Medical Perspectives on the Quest for Immortality

**DOI:** 10.1007/s10943-022-01591-9

**Published:** 2022-06-11

**Authors:** Ernst R. von Schwarz, Miguel Franco, Nathalie Busse, Sofiia Bidzhoian, Aubriana Angel Schwarz, Laurent Cleenewerck de Kiev

**Affiliations:** 1 Southern California Hospital Heart Institute, Culver City, CA USA; 2 Cedars Sinai Medical Center, Los Angeles, CA USA; 3 University of California Los Angles (UCLA), Los Angeles, CA USA; 4 University of Riverside (UCR), Riverside, CA USA; 5 Euclid University, Washington, DC USA

**Keywords:** Immortality, Religion, Longevity, Anti-aging, Christianity, Soul, Medical perspective

## Abstract

In the daily practice of medicine, health care providers oftentimes confront the dilemma of offering ‘maximum care’ based on available technologies and advances versus ethical concerns about futility. Regardless of cultural backgrounds and differences, most human beings aspire to an illness-free life, or better yet, a life lived with utmost quality and longevity. On account on ongoing advances in science and technology, the possibility of achieving “immortality” (a term used as a metaphor for an extremely long and disease-free life) is increasingly perceived as a realistic goal, which is aggressively pursued by some of the world’s wealthiest individuals and corporations. However, this quest is not taking place in a philosophical or religious vacuum, which is why we attempt to evaluate the current state of knowledge on religious beliefs revolving around immortality and their alignments with today’s medical advancements. The literature searches were performed using relevant databases including JSTOR and PubMed, as well as primary religious sources. Most religions present longevity as a blessing and believe in some sort of immortality, afterlife or reincarnation for the immortal soul. The quest for immortality beyond life in a “body of death” remains consistent with access to medical care and the legitimate possibility of achieving longevity—as long as certain ethical and religious parameters are preserved.

## Introduction

The cell is immortal. It is merely the fluid in which it floats that degenerates. Renew this fluid at regular intervals, give the cells what they require for nutrition, and as far as we know, the pulsation of life can go on forever. (Dr. Alexis Carrel, 1873–1944).

Advances in medical science have resulted in the possibility to delay certain processes of aging in many societies today, with the result that life beyond the usual life expectancy of seventy to 80 years could become an achievable goal in the near future. The imminence of this new horizon prompts us to consider the moral, philosophical, ethical and religious implications that it entails, especially for health care providers.

At present, the world largest companies such as Apple or Google as well as visionary individuals such as the CEO of Amazon, Jeff Bezos, are funding hundreds of millions of dollars on Silicon Valley Biotech companies to extend life through “cell reprogramming technologies” for the purpose of antiaging and ultimately, to achieve *immortality*. This purely scientific and technological quest is generally taking place in a materialistic and a-religious worldview. The contemporary revival of the alchemists’ quest for the philosophical stone should remind us that alchemy was a scientific idea coupled with a highly spiritual mindset (Principe, 2011). Yet currently, the discrepancy between personal faith and scientific advances seems to accumulate in the continuous quest for immortality among all cultures and religions (Brookhiser, 2007). From a physician’s point of view, it is oftentimes difficult to deal with patients’ requests and expectations on the possibilities of advanced medicine on one hand while facing incurable and deathly diseases on the other hand.

In the present article, we evaluate the philosophical, religious and medical perspectives on longevity and immortality in order to clarify some of the issues health care providers are confronted with by dealing with a possible collision between sciences and faith both in a professional and ethic context. Does the popular quest for immortality create a conflict for health care providers? Is there a conflict between religious faith and modern science?

## Methods

A literature search was conducted to evaluate the religious perspectives on immortality with regards to the major religions, namely (in chronological order) Hinduism, Judaism, Buddhism, Christianity, and Islam based on sacred scriptures. In addition, the medical perspective of current knowledge on immortality was assessed. Furthermore, philosophical aspects on immortality are briefly reviewed.

Various databases were analyzed such as JSTOR and PubMed, but also electronic versions of significant sacred texts including the Bible, the Bhagavad Gita, and the Quran. Search keywords included: immortality, religion, longevity, anti-aging, Hinduism, Buddhism, Christianity, Judaism, Islam, transhumanism, and faith.

## Study Limitations

The data gathered were product of the research and study of the available literature; it does not represent the view of any church or official religious societies. The present expositions represent a personal opinion of the authors. There is no claim to completeness due to the extensive implications on the study of religion. In that context, the focus on five major religions without taking other faiths into account constitutes the main study limitation.

## Results

A total of 75,595 scientific papers, articles and book chapters were found. In PubMed, the search term “immortality” revealed 25,671 hits, but the majority of the publications constitute studies about cell biology, telomerase shortening, protein markers of cellular immortality and cancer, among others.

In JSTOR, the search terms “immortality and religion” revealed 49,918 results; most of these articles represent descriptions of specific religious perspectives.

We evaluated the medical, philosophical and religious aspects of immortality, based on accordingly selected published data (Table 1).

**Table 1:** Perception of Immortality

Religion	Perception of immortality
Buddhism	The belief of reincarnation until reaching *Nirvana*
Christianity	Resurrection from death, immortality of body and soul in heaven or hell
Islam	Resurrection and retaining one's personhood and individuality regardless of whether or not the body is altered
Hinduism	Soul is immortal and reincarnation of soul into another physical body
Medical Perspective	Regenerative Medicine, anti-aging trials, enhancing quality of life while also yearning for longevity

### Philosophical Perspectives

From a philosophical point of view, there appears to be a difference between *eternal life, immortality*, and *everlasting life*. *Everlasting* is related to time, meaning there might be an end of everlasting even though it might be in a far future, considering the term “last” means “related to time”. In contrast, *eternal* life is independent on any concept of time and represents the timeless being of life.

The notion of the eternity of the soul stems from ancient Greek philosophers such as Pythagoras, Aristotle and his disciple Plato, who believed in the eternity and transcendence of soul (Jones, 2009).

In his essay on the *Theory of Soul*, Plato describes a duality of two separate entities: body and soul (Lorenz, 2009). The latter is considered immortal, incorporeal, above and beyond the visible, eternal occupant of our being, while the first is described as mortal, perishable, and disposable. According to Plato, the body unavoidably degenerates whereas the soul or the spirit, at the end of life, is set free from the prison of the body to live forever in the “world of the forms”, an ethereal and invisible only indirectly accessible concept in this world for those capable of higher thinking (Long, 2003). The dualistic approach of the Greek philosopher tended to look down on the body (being made of matter) as a reformatory for the soul and not the proper object of one’s eternal concerns. At the same time, the pragmatic Greeks highly valued not only physical beauty but also the quest for medical knowledge and medical ethics. Hence, Hippocrates—the acclaimed “father of medicine”—was credited by the disciples of Pythagoras of allying philosophy and medicine. His wholeness approach meant that body and soul were intimately interconnected, and that bodily health including longevity was a sign of spiritual well-being.

### Religious Perspectives

#### Hinduism

In Hinduism, the concept of immortality revolves around the mind and soul. The immortal soul is understood as experiencing a cycle of births and rebirths as it traverses through various physical bodies to progress on its endless journey (Dowley, 2018). While almost all belief systems consider time as linear, Hinduism perceives time as a cycle rather than a merely linear progression. A macrocycle (yuga) lasts several millions of years whereas a microcycle (samvatsara) is limited to a person's lifetime. The great Yuga Cycle (time cycle) is generally arranged in four phases, namely starting from Krita Yuga, Treta Yuga, Dvapara Yuga, and Kali Yuga (Rani, 2016). Hinduists believe that every living being will be reborn carrying a different name and taking on a different shape while progressing (or regressing) throughout a ‘cosmic accounting book.’ This cosmic accounting book has essentially two entries: – 1. the depositing credit people are born with, and 2. possessions (material and immaterial in nature) individuals accumulate or lose in the current lifetime. Hinduists perceive stories of people being extraordinarily talented in a particular field from early childhood itself according to this karmic method of accounting. In essence, the souls remain the same while its physical expression changes. The Hindu religion, civilization as some might state, is found in numerous works of literature in which the Vedas, Upanishads and two great epics (Ramayana and Mahabharata) carry a central theological authority (British Broadcasting Corporation: The Nature of Human Life in Hinduism). In the Bhagavad Gita, many verses describe the soul as being immortal, for instance:

na jāyate mriyate vā kadāchin

nāyaṁ bhūtvā bhavitā vā na bhūyaḥ

ajo nityaḥ śhāśhvato’yaṁ purāṇo

na hanyate hanyamāne śharīre

This verse translates to: the soul is neither born nor does it ever die, nor having once existed, does it ever cease to be. The soul is without birth, eternal, immortal, and ageless. It is not destroyed when the body is destroyed (Fosse, 2007).

However, there is also a concept of possible embodied immortality (the state of the gods) with amritam is being considered an antidote to death. It is described as an elixir drunk by gods that granted immortality (Sundararajan et al., 2003).

In the Katha Upanishad, the famous conversation between Nachiketa (a teen boy) and the god of death Yama states: *The all-knowing Self was never born, nor will it die. Beyond cause and effect, This Self is eternal. When the body dies, the Self does not die (*Whitney, 1890*).*

Another principal Upanishad (Brihadaranyaka) teaches: “When a lump of salt is thrown into the water, it dissolves and cannot be removed even though we can taste the salty water. So, the separate Soul dissolves in the sea of pure consciousness, infinite and immortal. Separateness comes when we identify the Self with the body; when this physical identification dissolves, the Self is no longer separate.” The Hinduist belief opposing the identification of the self with the body does not reflect the condemnation of blessed embodiment or the desire for longevity in general. Hinduism acknowledges the concepts of Chiranjeevee, which translates to “a long-living person” (of which there are eight examples). The cycle (samvatsara) of life and death is endless until one finds liberation (Moksha) by following the Hindu way of life. Until the elusive Moksha is attained either by good karma or nirvana, life goes on between birth and death, ideally a life of utmost spiritual and physical quality earned in part from past lives but also technological progress (Inbadas, 2017).

#### Hamsa Upanishad and Longevity

In their search for immortality, many sages and hermits practice different forms of yogic life, including Hatha Yoga and Kundalini Yoga. The central idea behind those practices is to control the breathing rate. Hamsa translates to “That I am” meaning “I am the same as the universe” (Rani, 2016). The average human performs 21,460 breaths daily (15 breaths/minute × 60 min/hour × 24 h/day). There was a quest to decrease the breathing rate by observing one’s breaths to attain longevity and possibly—immortality. This desire to lower the breathing rate and cardiac activities to lengthen the lifespan is consistent with modern science. After adapting to yoga and meditation, improvements in biomarkers of cellular aging and longevity were demonstrated recently (Bushell & Theise, 2009).

Indeed, the practice of slow breathing appears to optimize many physiological parameters of the respiratory, cardiovascular, and autonomic nervous systems, leading to longevity (Tolahunase et al, 2017). These practices could be a starting point to prove how yoga and meditation may delay aging. In the case of Hinduism, it is clear that the theological focus on the immortal soul is not incompatible but rather congruent with the quest for longevity, as long as the extended lifespan is used to perform good deeds and life a life consistent with the Hindu religion.

### Judaism and Christianity

Both Judaism and Christianity share the Old Testament as a foundational theological text. Genesis, in particular, presents man (Adam) as a functional unity of body and breath (or spirit), “a living soul.” Physical aging and death are presented as the consequence of sin, but no immortality or survival of the soul is presented in these early texts, or anywhere else in the biblical Scriptures.

The genealogies found in Genesis provide extraordinary lifespans for early humans, with chapter 6 suggesting a radical change in this situation after the flood. Still, these passages indicate the blessed are shown to experience a lifespan of over a hundred which is high by modern standards but short in comparison to previous generations, according to the texts. Obviously, not all scholars take these numbers literary or even as providing any reliable information on lifespans.

However, theologians agree on the development of a belief in some form of bodily or spiritual survival, which is why King Saul is convinced that the spirit of a dead prophet can be summoned. The concept of a resurrection of the body (possibly hinted at by the care taken for human remains) becomes clearer in the late Old Testament (intertestamental era), for instance in Daniel 12:

And many of them that sleep in the dust of the earth shall awake, some to everlasting life, and some to shame and everlasting contempt.

And they that be wise shall shine as the brightness of the firmament; and they that turn many to righteousness as the stars for ever and ever (James, 1611).

The Christian tradition continued to emphasize the goodness of the body and the hope of resurrection of the body as a “spiritual body,” the exact nature of which was the object of debate. At the death of the body, the Jewish belief was maintained that a relatively or conditionally immortal soul would survive and find rest in place of various pleasantness until the ‘end of the age’ (and with it the general resurrection and final judgment).

Although Christian authors such as St. Paul and St. Ignatius of Antioch express a certain eager to depart from the body and “be with the Lord,” nothing in these teachings would oppose the human desire, expressed in the Psalms, to live a long life and “see your children’s children.” Coupled with the adoption of such books as Sirach which offers a high praise of doctors (Sirach 38), this makes the quest for a longer life span with a righteous intention compatible with most expressions of the Christian faith today. However, the quest for actual immortality or to replace the failing human body with artificial intelligence should be recognized as at odds with the fundamentals of the Christian hope.

### Buddhism

Buddhism may be viewed as controversial compared to other beliefs as it lacks some common characteristics of most major religions. However, Buddhism does have its roots in India as well as the Hindu belief in karma and reincarnation along with the desire to escape the endless cycle of bodily birth, death, and reincarnation (Arnold, 2008). At its core, the Buddhist school of thought preaches the human ability to achieve ‘divine’ attributes, such as immortality and omniscience. At the same time, Buddhism does not present a ‘ruler god’ that must be served, pleased or praised. Instead, Buddhists consider Siddhartha Gautama (“The Buddha”) the central representation of a man who reached Nirvana and shared his teachings with his followers (JAMA, 1909). This process is described in the Tipitaka, the sacred canon of Theravada Buddhism, based on the seven main Buddhist teachings:

1. The Four Noble Truths.
2. The Noble Eightfold Path.
3. No killing. Respect for life.
4. No stealing. Respect for others' property.
5. No sexual misconduct. Respect for pure nature.
6. No lying. Respect for honesty.
7. No intoxicants. Respect for a clear mind.

Hence, the ultimate object of life is to acquire freedom from the limitations of the material world by substituting volitional for sensory consciousness (Saisuta, 2012). Because Buddhism presents desire (notably bodily desires) as the root cause of suffering and sensory entrapment, the medical and overly non-spiritual quest for bodily immortality is hardly in conformity with this fundamental proposal. However, Buddhism as a worldview and philosophy of life takes many forms, many of which are fully compatible with the quest to alleviate suffering and maximize the human potential in order to contribute to the enlightenment of the world.

### Islam

Islam is based upon the Jewish and Christian narratives as alluded to or recounted in the Quran. Muslims have a very tangible vision of paradise rewarding good deeds performed during one’s physical lifetime. The soul’s residence in a human body is understood as a tremendous blessing (with the philosophy equally applying to Judaism and Christianity): the Quran reiterates the ancient story about the arrival of the first human being on earth. According to the scriptures, the angels were told to offer homage to this new creation (by bowing down or prostrating), an act which led to the rebellion and fall of some of them. This rather physical perspective underlies a “theology of the body” (rooted in Catholicism) which puts emphasis in view of the quality of human life in the present moment (Talal, 2012).

In view of the progressive development of the Islam in the seventh century, this understanding can be associated with the advancement of scientific knowledge and a less traditional way in which medicine was perceived. For instance, Ibn Al Nafis, a Muslim physician, described pulmonary circulation 300 years before William Harvey (who is widely accepted to having recognized the same physiological parameters first) was born (Al-Ghazal, 2007). Abu Al Qassim AlZahrawi’s elaboration became the standard medical resource for European universities. Accordingly, Al Razi and Ibn Sina (Avicenna) were known as pioneers in the field of medicine. The holistic approach used in modern medicine was initially described by these Muslim physicians. Islam sees great value in advocating for further development in science and acknowledging the art of the human body. In the Quran, Surat Fatir 28 states that those who fear Allah the most are those who are the most knowledgeable with reference to general knowledge and the sciences (Amr & Tbakhi, 2007). Historically, great emphasis was placed on the acquisition of cures for all disease based upon the Quranic postulating that “Allah has not sent any disease without sending a cure for it” (Khan, 2013). This principle is further supported by the hadith of Abu al-Darda: “The Prophet said: Allah has sent down both the disease and the cure, and He has appointed a cure for every disease, so treat yourselves medically, but use nothing unlawful” (Dawud & Ash’ath, 2008).

On the other hand, in the proper meaning of the word immortality, the Quran clearly states that every human being shall taste death in one manner or another (Al-Ghazal, 2007). This teaching is in concordance with the Christian scriptures: every human being is bound to taste death: and one shall receive his or her rewards in full on the Day of Resurrection.

The idea of a legitimate quest for health and longevity with the reminder to (1) “use nothing unlawful” (in Islam, this may be interpreted as not using products derived from pigs) (Dawud & Ash’ath, 2008) and (2) avoid being distracted from pursuing one’s religious quest and duties (Effendi, 1960) is not limited to concepts of Islam but also applies to other religious traditions.

### Medical Perspectives

The medical sciences exist as a result of humanity’s desire to preserve life and health. One of the most fascinating vestiges of civilization resides in the remains of a hominid with data on a healed femur fracture (Blumenfeld, 2020). We distinguish ourselves by caring to heal the wounded, help the needy, and live together in society*.* The structure of our intellect as well as scientific and technological advances led us to systematize our practices, establish codes and treaties to define good practice in any discipline.

The Hippocratic Oath, written between the fifth and third centuries BC command the physician to show “utmost respect for human life from its beginning,” beginning with the “*Primum non nocere*” (“first do no harm”). In a modified form, this oath is taken by physicians around the world and encourages us to preserve life and its quality, which aligns with the concept of delaying aging and pursuing immortality (National Institutes of Health, 2012).

The concept of “health span”, meaning prolonging life in regard to quality of years lived rather than just surviving the years left to live, is stressed through various physiological parameters, as described below (Crimmins, 2015). Ancient and modern medicine have identified many approaches to maximize life in quantitative and qualitative terms. For instance, calorie restriction before the onset of old age (i.e., before 60 years) could delay aging through various negative/positive feedback mechanisms, thereby delaying cardiovascular aging, decreasing the risk of cancer and other metabolic disorders, enhancing the quality of life, and leading to longevity. In addition, there are reports of some drugs such as Metformin, a diabetes drug, and Rapamycin, an antifungal antibiotic drug, that could potentially serve as “anti-aging” medications due to their anticancer properties and documented evidence of prolonging life through the process of suppressing progressions of aging (geroconversion) in experimental settings, in which animal studies suggest that consuming the medication prior to the onset of illness reveal superior benefits in terms of mitigating the effects of aging (Blagosklonny, 2019; Soukas et al, 2019). The use of anti-aging remedies, when done in a subclinical phase, has resulted in convincing evidence that it is possible to enhance human life in terms of quality and lifespan (Blagosklonny, 2012).

Implementing lifestyle modification and adopting a ‘primordial prevention system’ while decreasing risk factors could reduce cardiac death by 90% and eventually prolongs life-expectancy by ten or more years. Furthermore, in addition to medical treatment, it is now recognized that psychological, nutritional, and social/family support can extend the longevity of an individual's mortal years to achieve paramount life years (Eissenberg, 2018; Mishra, 2016; Russo et al, 2017).

Certain plant-based supplements such as adaptogens, bacopa monnieri, curcuma longa, emblica officinalis, ginkgo biloba, glycyrrhiza glabra, and panax ginseng and plant-based metabolites such as polyphenols, carotenoids, vitamins C and E possess anti-inflammatory, antioxidant, and anticancer properties that might affect lifespan (Dhanjal et al, 2020). These supplements are within reach to a vast segment of the population if the desire to pursue such a ‘longevity promoting lifestyle’ is strong enough.

The concept of “inflammaging” is a new paradigm according to recent studies on aging that is yet to establish concrete inferences and protocols (Sanada et al., 2018). This process is described as a chronic and persistent phenomenon that progresses along with age through an inflammatory cascade, ultimately exhausting the body's defense system. Recent studies using mesenchymal stem cell therapy have shown promising results on immune system modulation to a level where an illness-free state could be reached with the mere properties of tissue regeneration and immunomodulation, thus supporting the existence of inflammaging (Lee & Yu, 2020).

A study in 2006 attempted to replace diseased organs in the body through the use of pluripotent stem cells. These induced pluripotent stem cells demonstrated growth properties and a morphology similar to embryonic stem cells displaying embryonic stem cell marker genes. The idea behind is to reprogram a cell to regain an immature status before developing into any desired body tissue, demonstrating that cells can be manipulated through *genetic reprogramming* to avoid senescence (Takahashi & Yamanaka, 2006).

Regenerative medicine by use of stem cells is one of the most promising advances in modern medicine, which involves the use of cellular and/or acellular products to stimulate metabolic pathways that normally tend to deteriorate with aging, and to replace dead tissue (von Schwarz, 2022).

## Discussion

When discussing immortality from a philosophical, theological and medical perspective, we may ask the following three questions:

1. Is immortality biologically possible?
2. Do patients perceive advances in medicine as a way to cheat and avoid death which then replaces any faith in religious ideas and traditions?
3. What is the physician’s role in this context?

### Is Immortality Biologically Possible?

Based on the current status of biotechnology and advances in regenerative medicine and cell reprogramming, it appears to be technically possible to create cells in a state where either aging processes are significantly delayed or completely prohibited in the near future. However, does it mean that the immortality of the human would be possible in our lifetime? Even though we do not have the answer, it is more unlikely than likely that biotechnology could advance to create immortality in a sense of abolishment of aging and death from natural causes. If we refer to biological, natural immortality—in contrast to immortality after someone died in the sense of spiritual immortality—true biological immortality appears too far of reach for our lives at the current time. However, biotechnology advances further in techniques to replace degenerated tissues and to halt processes of aging, which will result in a prolongation of life.

### Do Patients Perceive Advances in Medicine as a Way to Cheat and Avoid Death Which Then Replaces any Faith in Religious Ideas and Traditions?

Biological therapy should not be considered as replacement to faith. According to Christianity, there is no controversy between faith and advancement in sciences despite the controversial history between religion and science.

Advances in biotechnology and natural sciences do not make faith obsolete. While science explores how things happen, religion and faith still explore why things happen, which still has not been answered—including the creation.

Modern biotechnology does not replace religious beliefs in most religions including Christianity, Judaism and Islam as a whole for the following reasons:

1. Biology is not able to replace faith as a way of life and living, as a guide through hard times, as something giving hope for all believers.
2. Computed technology does not replace human consciousness, thoughts, abstract thinking, reasoning, conscious decision-making, or emotional feeling such as happiness, grief, hope or love.
3. Science is able to explain how things work but fails to explain why things happen.
4. Science is limited in its ability to explain the unknown and the unexplainable.
5. Science is constantly changing while faith remains unchanged over thousands of years.
6. Christian faith and the Catholic Church as well as most other religions do accept modern sciences including Darwin’s theory of the evolution.
7. Biblical writings are meant to be understood symbolically rather than factually.

### What is the Physician’s Role in this Context?

Medical providers increasingly report on increasing interest in the quest for longevity and immortality, possibly due to the emerging perception of the introduction and availability of both medical and technological breakthroughs.

Every day, doctors treat people of faith by preventing illness, treating the symptoms and attempting to cure disease. At the same time, present-day medical science is being combined with technological advances to pursue the same and ultimate goal of permanence with optimal quality of life through regenerative medicine.

It remains important to engage the philosophical considerations of an immortal soul which according to religious traditions and beliefs might be reunited to our body in an afterlife after our earthly existence. Faith has proven to be an essential part of human life throughout history, and several studies (including one of our own) have shown that hospitalized patients sustained by any religious belief have a better outcome than those who do not believe in anything transcendent (Naghi, 2012).

People of faith, patients and doctors, are becoming increasingly aware of promising developments related to our understanding of aging and cellular death as well as the potential of prolonging life by either manipulating age-related processes by genetic programming or by using regenerative medicine instead of reactive medicine to repair damage using stem cells and other methods.

From a physician’s point of view, we need to remind ourselves that at the current time, these promising advances in science and technology are seldom applicable to practical medicine. In other words, patients with underlying progressive malignancies, such as metastatic cancer or end stage heart failure, are still going to die, and physicians should internally and externally admit their inability to defy death.

What is the physician role in this context? At this point, as a health care provider or physician, we are obliged to continue providing maximum care but also consider futile care in cases of end-stage diseases without options and in end-of-life situations and be realistic with regard the expectations of patients or their family members. Medicine is here to alleviate symptoms and pain and restore function and sometimes to prevent death and prolong life; however, physicians do face limitations in certain conditions where medical therapy might only be able to offer comfort care measure at end-of-life situations.

### Outlook

All unconscious cognitive processes such as thoughts, perceptions, memories, emotions, will, and imaginations could be personified as the indivisible soul (or mind or consciousness) (Pandya, 2011). The physical body (regardless of how it relates to the mind) is the one that tumbles into ailments sooner or later in our lifetime, whether through degenerative (age-related) processes leading to chronic diseases, or through infections, traumata, or via cellular mutations leading to malignancies (which also might be to some degree age-related). However, the soul/mind does not always suffer from any physical injury and many hope as proposed in traditional Greek philosophy and in many religions that it may continue to exist, indefinitely (Mellert, 1951). The quest for bodily immortality is as old as humanity, likely arising from the incomprehensiveness of ideas and reasons behind our existence, observed experiences of death among others, the fear of dying, and the cruel anticipation of absolute cessation of our existence thereafter. Modern medicine is evolving and attempting to offer biological solutions to the human quest for permanence of existence. Religions around the world are increasingly having to deal with this development, while the global economy is also faced with related dilemmas.

A core issue is whether the human race is capable of reprograming cells to an embryonic stage where age is just a number and regeneration could be a possible answer to all ailments ever witnessed in the life on this planet. Does this possibility negate faith, religious beliefs, and even our conscious and unconscious processes of living life morally good in order to condition ourselves for the afterlife?

Clearly, one significant difference between the religious and scientific approach is that the former refers to a concept closer to “eternity” as timeless life, an ‘unprovable fantasy’ from a purely scientific perspective, whereas with science we are aiming to achieve delayed aging and a prolonged lifespan, at the risk of sidestepping previously held ethical boundaries.

The common denominator between religions on this topic tends to be the spiritual or divine nature attributed to immortality as an afterlife objective. While natural longevity and prolongation of life are mentioned in sacred texts, such a quest is rarely present as relevant. This idea of eternity, a never-ending life, or an afterlife in could be conjugated as a notion we could call “permanence”. Depending on the religion, permanence could be achieved either by the spirit, soul, or a resurrectional body. The spiritual energy or soul is often understood as an “epitome” that works as an avatar for the conscious mind to achieve *permanence* regardless of the state of the body.

Therefore, ongoing advances in technology, science and modern medicine should be seen as complementary rather than contradictory to one’s faith in an everlasting existence, either on the soul or resurrection of the human being after our natural death.

This quest to achieve longevity through both science and technology may also turn into a new form of ‘faith’ as the possibility to achieve this goal remains unanswered.
